# Environmental catastrophes, climate change, and attribution

**DOI:** 10.1111/nyas.14308

**Published:** 2020-02-11

**Authors:** Elisabeth A. Lloyd, Theodore G. Shepherd

**Affiliations:** ^1^ Department of History and Philosophy of Science and Medicine Indiana University Bloomington Indiana; ^2^ Department of Meteorology University of Reading, Reading, United Kingdom

**Keywords:** environmental catastrophe, ecology, ecosystems, climate change, extreme weather events, attribution

## Abstract

In our discussion of environmental and ecological catastrophes or disasters resulting from extreme weather events, we unite disparate literatures, the biological and the physical. Our goal is to tie together biological understandings of extreme environmental events with physical understandings of extreme weather events into joint causal accounts. This requires fine‐grained descriptions, in both space and time, of the ecological, evolutionary, and biological moving parts of a system together with fine‐grained descriptions, also in both space and time, of the extreme weather events. We find that both the “storyline” approach to extreme event attribution and the probabilistic “risk‐based” approach have uses in such descriptions. However, the storyline approach is more readily aligned with the forensic approach to evidence that is prevalent in the ecological literature, which cultivates expert‐based rules of thumb, that is, heuristics, and detailed methods for analyzing causes and mechanisms. We introduce below a number of preliminary examples of such studies as instances of what could be pursued in the future in much more detail.

## Graphical abstract

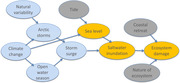

## Introduction

It is widely recognized that climate change has the potential to induce environmental catastrophes, such as ecosystem collapse.^1^ It is also widely recognized that many impacts of climate change occur through extreme weather events.^1^ This is because human and natural systems always have a certain degree of resilience to fluctuations, and it is the fluctuations outside the resilience boundaries of the system that lead to detrimental impacts, or even to system collapse. These considerations motivate the question of how climate change may affect ecosystems through extreme weather events. By *extreme weather events*, we mean any extreme fluctuation in the state of the physical climate system (including the ocean) that has a component of natural variability. The concept thus includes both short‐term events, such as tropical cyclones, and long‐term events, such as multiyear drought, as well as compound events.^2^

The term *extreme* is generally understood in one of two ways. The first is as a statistical extreme, that is, defined in terms of rareness. This is the usual approach taken in the climate science literature,^3^ where extremes are typically defined as the occurrence of the value of a quantity either above a threshold near the upper end of its climatological distribution, or below a threshold near the lower end of its distribution. For example, for temperature, a warm extreme might correspond to values in the upper 5% of the distribution, and a cold extreme to values in the lower 5%. The distribution is generally defined on the basis of a particular reference period. The second way in which *extreme* is understood is in terms of impact. A rare event may not necessarily be impactful, and an impactful event may not necessarily be rare. An example would be tropical cyclones, which are invariably impactful yet are not rare in certain parts of the world at certain times of year (even if they only infrequently affect any particular location); indeed, a tropical cyclone season without a tropical cyclone would itself be a rare event.

**Figure 1:**
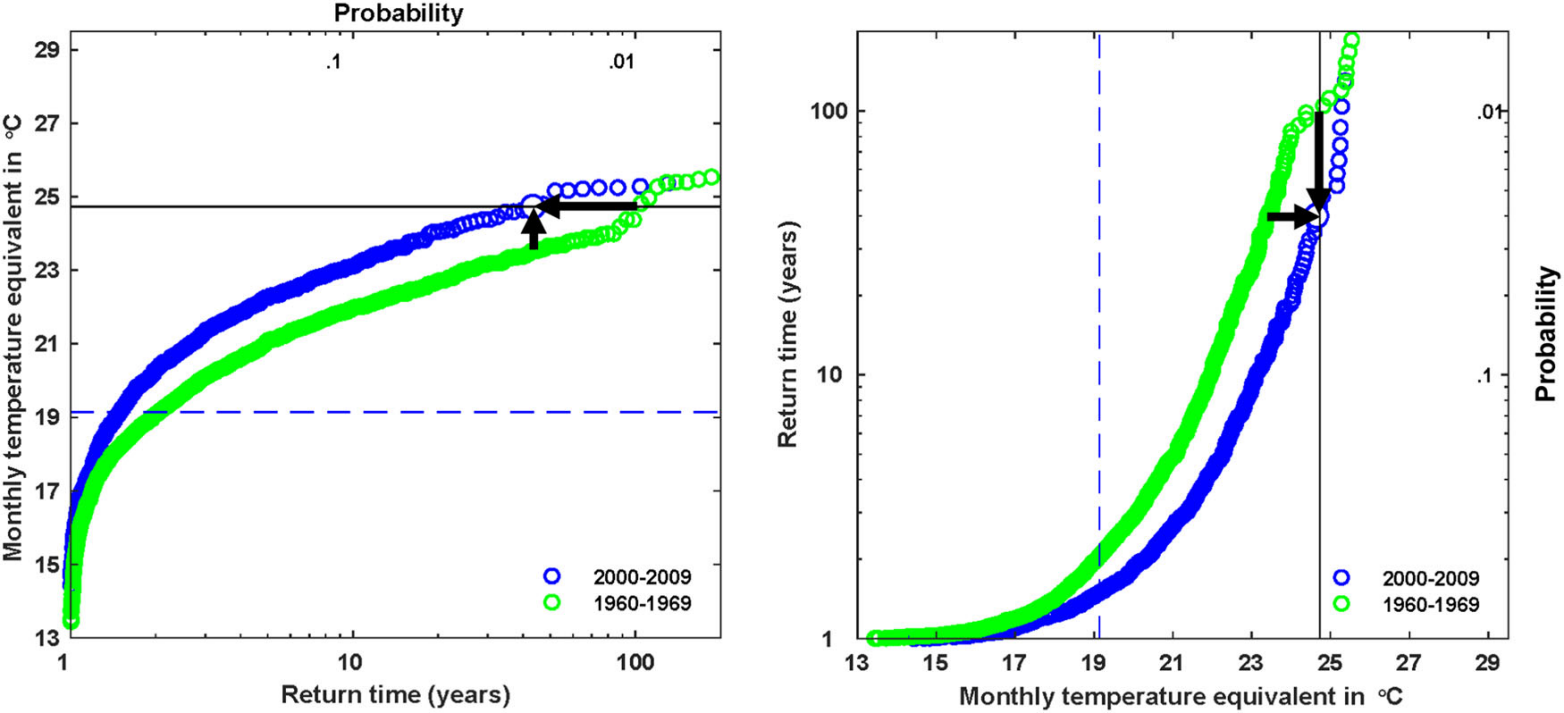
The effect of anthropogenic climate change on July heat waves over western Russia, motivated by the extreme heat wave of summer 2010. In both panels, the actual event magnitude is indicated with the solid black line and the July mean temperature during the 1960s with the dashed blue line, together with modeled estimates of the likelihood of exceeding a particular temperature threshold for both 1960s (green) and 2000s (blue) conditions, in terms of either probability or return time. The left panel shows magnitude versus probability, while the right panel shows probability versus magnitude. The two black arrows in each panel point to the observed event in the factual calculation. Anthropogenic climate change is seen to have increased both the magnitude and the probability of the heat wave. From Ref. 2, and adapted from Ref. 16.

The statistical definition of *extreme* is attractive to climate scientists because it appears to avoid the subjectivity that is inherent in any impact‐based definition. Under certain conditions, it can also allow the application of a branch of statistics known as extreme value theory, which is a way to estimate the likelihood of very rare events on the basis of a limited set of data. However, the need to create a statistical distribution in order to compute probabilities is necessarily a drastic simplification of reality.^4^ Most published studies of statistical extremes are univariate, and although there is a growing awareness of the importance of estimating risk from compound events,^5^ the ability to treat multivariate situations statistically remains limited.^5^, ^6^ Consider again the case of a heat wave. For temperate regions of the planet, extreme summertime temperatures are often associated with what meteorologists call “atmospheric blocking” flow conditions, where the air becomes stagnant.^6^ These conditions are also associated with a lack of precipitation and possibly with extreme air quality conditions. Under climate change, if exceeding a particular temperature threshold that used to be rare now becomes more common, as is invariably the case when considering a fixed temperature threshold (see the discussion of Fig. 1 below), then it would not necessarily be associated with such extreme blocking conditions, and thus with the other aspects of the extreme event. In which respect, then, is it sensible to talk about the likelihood of such a heat wave “event” changing under climate change? The situation is even more complex in situations with strong local effects and multiple causal factors, such as Superstorm Sandy, which resulted from a collision between a rogue tropical cyclone and a midlatitude weather system.^7^

The fact is that every extreme event is unique, and this is even more the case when the event includes effects on ecosystems. There is, therefore, a fundamental decision to be made as to whether one studies an event from a statistical perspective, aggregating over an inhomogeneous (i.e., not identically distributed) population of similar kinds of events, or from a singular or “case study” perspective, treating the event as unique. The singular approach identifies the different causal factors that played a role in an event and considers the outcome of counterfactual situations in which one or more factors were different, as in a forensic investigation. In most fields of scientific inquiry, both approaches can claim some kind of provenance. Randomized control trials are a statistical approach to attributing cause and effect in many contexts, but Cartwright argues^8^, ^9^ that they do not provide the standard of evidence they claim, and that singular causation is a perfectly sensible concept. For extreme weather events, Shepherd^4^ characterizes the statistical perspective as the “risk‐based” approach and the singular perspective as the “storyline” approach. Shepherd further emphasizes the complementarity of the two approaches, and that any particular study may combine elements of each.^4^

Singular causation may also be seen as conditional causation, where one or more factors are varied, and the others are regarded as contingent for the purpose of the analysis.^10^ In that respect, the causation is conditional on the state of the remaining factors. This concept is extremely useful when there is a high degree of uncertainty in the problem under analysis, as is generally the case when considering the impacts of climate change. In particular, since human and natural systems are often changing quite rapidly for reasons not directly associated with climate change, it is generally extremely difficult to make unconditional statements about the impacts of climate change because of these confounding factors. In the IPCC Guidance Document for AR5 on Consistent Treatment of Uncertainties (see Ref. 11), it is emphasized how the language of conditional findings (effects) provides a way to express confidence in the cause of an effect given the antecedent conditions, when confident statements about the cause of the antecedent conditions are not available. The legal equivalent is “ceteris paribus.”

These different approaches to causation and the construction of evidence are especially relevant when it comes to the question of whether a scientific analysis chooses to minimize the likelihood of a Type 1 error (a false positive or false discovery) or Type 2 error (a false negative or missed warning). By aggregating over an inhomogeneous population, statistical approaches are prone to miss the signal and conclude with a statement, such as “no effect detected,” which makes them prone to Type 2 errors. By contrast, singular approaches may miss consideration of a relevant causal factor, which makes them prone to Type 1 errors. Lloyd and Oreskes^12^ asked why it is that in climate change science it has been considered scientifically rigorous to preferentially guard against Type 1 errors, and advocate for a more active consideration of singular causation as a way of guarding against Type 2 errors.

In this paper, we consider both approaches to causation in the context of how climate change affects ecosystems through extreme weather events. We do so through a “logic of research questions” analysis,^13^ applied to a number of case studies of extreme ecosystem events. In general, the risk‐based approach asks the following research questions:

R1: How was the likelihood of the event affected by climate change?
R2: How was the magnitude of the event affected by climate change?

These two questions immediately require a deliberately approximate definition of what is meant by the event, because to enable statistical analysis, the event must be generalized in some fashion. Indeed, often the event that prompted the analysis is excluded from the subsequent analysis, and the threshold lowered, in order to avoid what statisticians call selection bias.^2^ By contrast, the storyline approach asks the following research questions:

S1: What were the relevant causal factors that led to the event?
S2: How might climate change have contributed to those causal factors?
S3: How might future climate change make a future such event even more impactful?

These questions take the event as given and allow a layered approach to attribution within a conditional framework. The conditionality of the attribution means that different storylines can be considered depending on the question being asked, for example, depending on whether one wishes to preferentially guard against Type 1 or Type 2 errors.

Before applying this analysis to our ecosystem case studies, we first review the concept of extreme event attribution in the weather context in which it first arose and consider one of the few end‐to‐end studies addressing the impact of an extreme weather event on the human environment.

## The concept of extreme event attribution

The field of extreme event attribution as applied to weather events has developed over roughly the last 15 years, and is now very active.^2^, ^14^ Because climate is generally understood to represent the distribution of possible states, the standard approach in climate science is to examine changes in the statistics of extreme events.^3^ However, there was a growing demand, at least in the media, for climate scientists to be able to make quantitative statements about individual extreme events, beyond a qualitative statement, such as “This kind of event is expected to become more likely as a result of climate change.” Stott *et al*.^15^ were the first to create such an analysis by using a single event (the 2003 European heat wave) to motivate an event class (average summertime temperature over a region encompassing Southern Europe and the Mediterranean basin exceeding its 1961–1990 mean by at least 1.6 °C), and then assess changes in that event class using standard statistical methods. In this way, the attribution question is turned into a climate question, which is not different in kind from any other sort of climate analysis.

The method is illustrated in Figure 1, from Ref. 16, for the case of the 2010 Russian heat wave. First, a climate model simulation is performed under present‐day (so‐called factual) conditions. Then, another model simulation is performed under historical (so‐called counterfactual) conditions, with a reduced amount of climate change (or perhaps none at all). The likelihood of exceeding a particular absolute temperature threshold (usually expressed in terms of a return time) is then plotted for both cases (blue and green points, respectively), and the difference between the two quantified. The difference between the factual and counterfactual conditions can be represented either in terms of a change in likelihood for a given temperature threshold, or a change in magnitude for a given likelihood. As pointed out by Otto *et al*.,^16^ the choice of perspective (likelihood versus magnitude) has a profound effect on the conclusion regarding the role of climate change. In particular, the change in magnitude is generally a small fraction of the total anomaly, reflecting the fact that natural variability is the main driver of the extreme event. In this case, because the region is smaller (western Russia), the time period is shorter (July only), and an index of atmospheric blocking is used as a covariate, the temperature anomaly is much larger than in the study by Stott *et al*.^15^ The change in likelihood is more striking than the change in magnitude and is usually the metric emphasized in such studies.

The above examples illustrate that the statistical approach to event attribution cannot avoid subjectivity, because the definition of the event is inherently subjective, for example, in choosing the beginning and ending of the event or the thresholds for the category, and can have a very strong effect on the outcome. For example, because of such ambiguity, two prominent studies of the 2018 European heat wave came to very different conclusions on the importance of climate change, with the one using a 3‐day event definition estimating a two‐ to four‐fold increase in likelihood,^17^ and the other using a 3‐month event definition a 30‐fold increase.^18^ Also, even for a given definition of the event, it is not clear how to estimate the full uncertainty of the calculation. The aleatoric (random) component of the uncertainty, which is associated with natural weather variability, can be determined by employing a sufficiently large model ensemble. This is generally done by using an atmosphere‐only model with prescribed sea‐surface temperatures, which can be run much more efficiently than a coupled climate model and thus can be used to generate a large sample size. However, the epistemic (systematic) component of the uncertainty is difficult to assess. One element is the choice of the atmospheric model used. A second element is the estimate of the anthropogenic component of the warming of sea‐surface temperatures, which is used to produce the counterfactual calculation. In practice, a number of different estimates are produced from different coupled climate models, and these are taken to represent a range of uncertainty.

**Figure 2:**
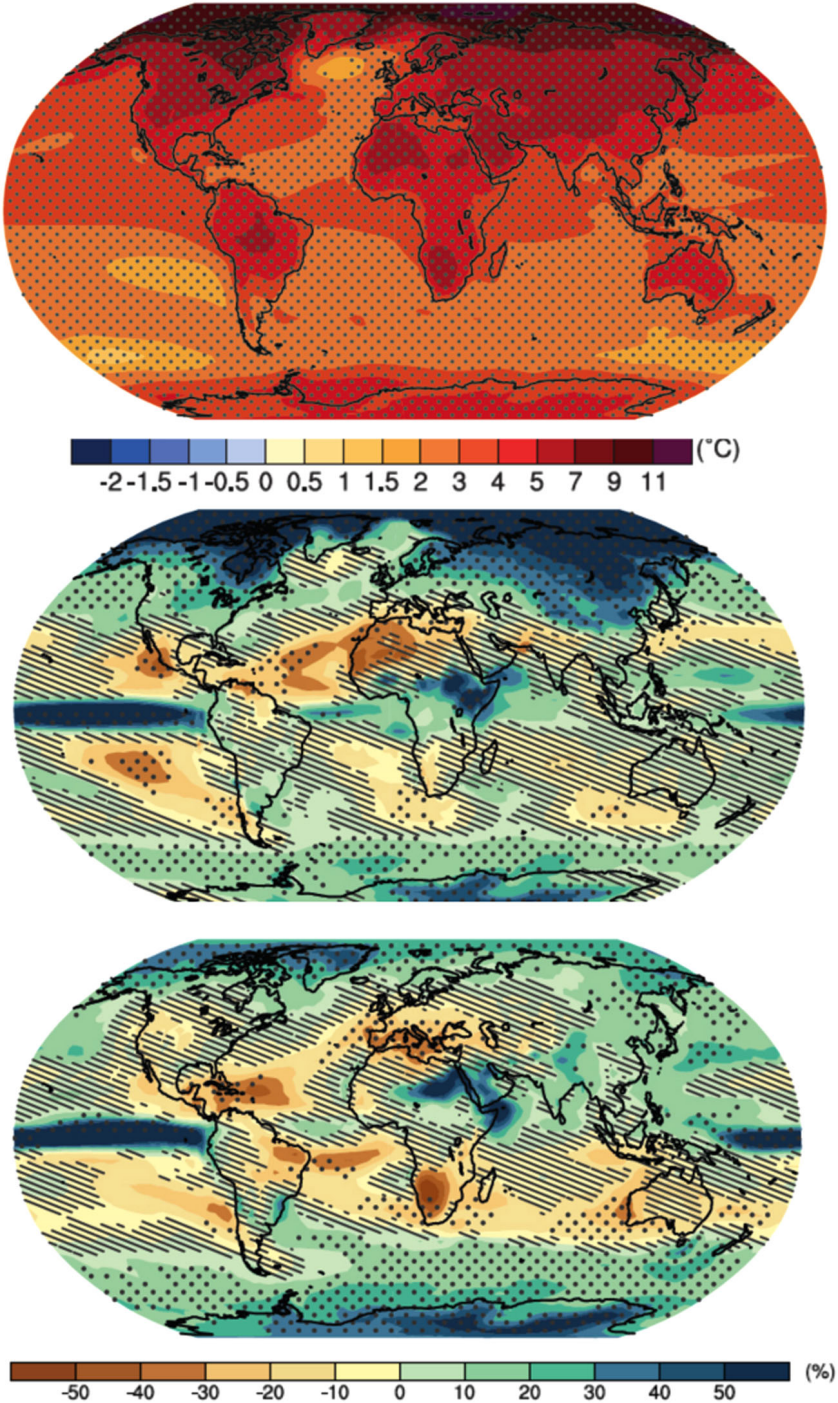
CMIP5 projections of changes in annual mean temperature (top) and in boreal winter and summer precipitation (middle and bottom) by the end of the century under the RCP8.5 forcing scenario. Stippling indicates where the model projections are robust, in the sense of agreeing on the sign of the change; otherwise, the models do not agree. Hatching indicates where the average model changes are small compared with internal variability, but this does not mean that individual model changes are small. Warming is robust over all land areas. Precipitation changes can be of either sign and are nonrobust over the regions and seasons discussed in our case studies. From Ref. 64.

While the epistemic uncertainty in thermodynamic aspects of climate change (e.g., warming of atmosphere and ocean, melting of ice, and sea level rise) is essentially quantitative, the epistemic uncertainty in dynamic aspects (e.g., storm tracks, persistent atmospheric circulation anomalies, and El Niño conditions) is often still qualitative, that is, with uncertainty in the sign and nature of the change.^19^ The difference is illustrated in Figure 2, where the changes in temperature projected by climate models are seen to be highly robust, but the changes in precipitation are often not, especially over the more populated land regions of the planet. Although it is true that a warm atmosphere can hold more moisture (a thermodynamic statement), this does not directly translate into precipitation because of the key role of dynamical processes, as Figure 2 indicates. In our case studies, we will often return to this figure. Given the very different levels of confidence concerning thermodynamic and dynamic aspects of climate change, there is much more confidence in the attribution of extreme weather events whose causal factors are more closely tied to global‐mean warming.^2^ This will be a recurring theme in our case studies.

**Figure 3:**
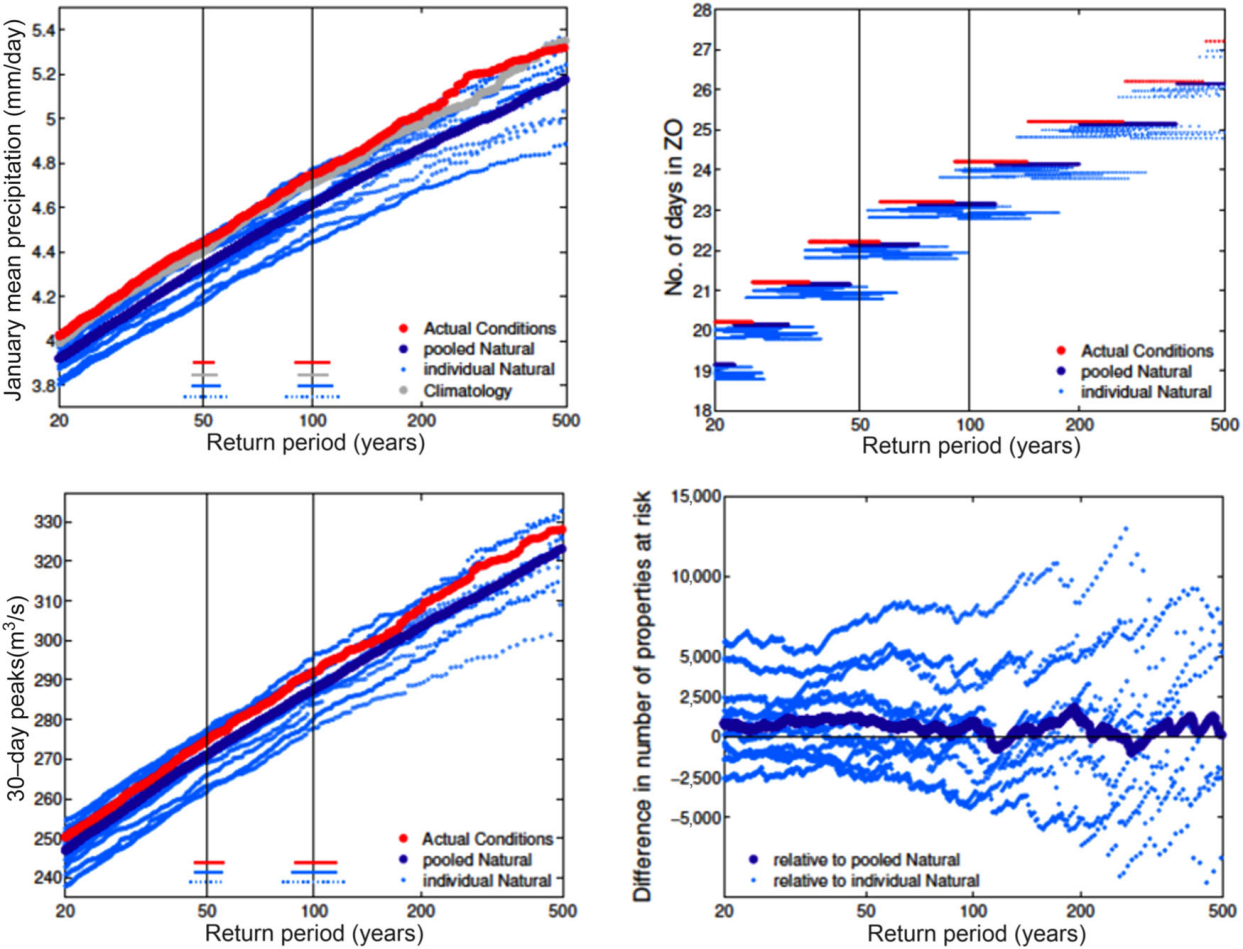
The modeled effect of anthropogenic climate change on wintertime flooding in the Thames Valley, motivated by the flooding in winter 2013/2014. Top left: January mean precipitation over Southern England. Top right: Return periods for a “stuck” jet stream, labeled “ZO.” Bottom left: Return periods for 30‐day peak flows for the Thames at Kingston, close to London. Bottom right: Difference in number of properties at risk of flooding as a function of return period. The estimates from the factual calculation are shown as a set of red points, and from each counterfactual calculation (using different estimates of the anthropogenic change in sea‐surface temperatures) as a set of light blue points, with the average shown in dark blue. From Ref. 20, with permission.

Most published studies of extreme weather events stop at the weather, for example, precipitation in the case of flooding. The issues become even more complex when the impacts of the extreme weather event are considered. An illustrative study is that of Ref. 20 on flooding in the Thames River valley in England. In the winter of 2013/2014, a series of storms hit southern England that caused very severe flooding and over £450M in insured losses. No storm had precipitation that was particularly intense, but the precipitation fell repeatedly in the same places. From a weather perspective, the proximate cause of the flooding was thus the “stuck” jet stream that led to the clustering of the storms in the same location. As shown in Figure 3, Schaller *et al*.^20^ studied a large ensemble of climate model simulations to determine a causal account of the extreme precipitation like that seen in this English winter, finding that the likelihood of a stuck jet stream was indeed a relevant causal factor. Note that there is no clear view from the climate science community concerning the expected response of the jet stream in this region to climate change.^21^ The different counterfactual calculations, based on different assumptions regarding the anthropogenic warming of sea‐surface temperatures, were taken as providing a measure of uncertainty. The estimates ranged from no discernible effect to an increased likelihood of a stuck jet stream and increased precipitation, which, treating the epistemic uncertainty in an aleatoric fashion, the authors interpreted as an attribution to anthropogenic climate change. They then applied the extreme precipitation to hydrological modeling of the Thames River flows. While the 30‐day peak flows still showed a tendency toward an increase, this did not propagate through to the number of properties at risk, which either increased or decreased depending on the counterfactual assumption. Schaller *et al*.^20^ conclude that flood risk mapping shows “a small increase in properties in the Thames catchment potentially at risk of riverine flooding,” within a substantial range of uncertainty. Importantly in our context, they also conclude that their study demonstrates the significance of explicit modeling of impacts, given the relatively subtle changes in weather‐related risks, when trying to calculate the effect of climate change in extreme environmental events.

**Figure 4:**
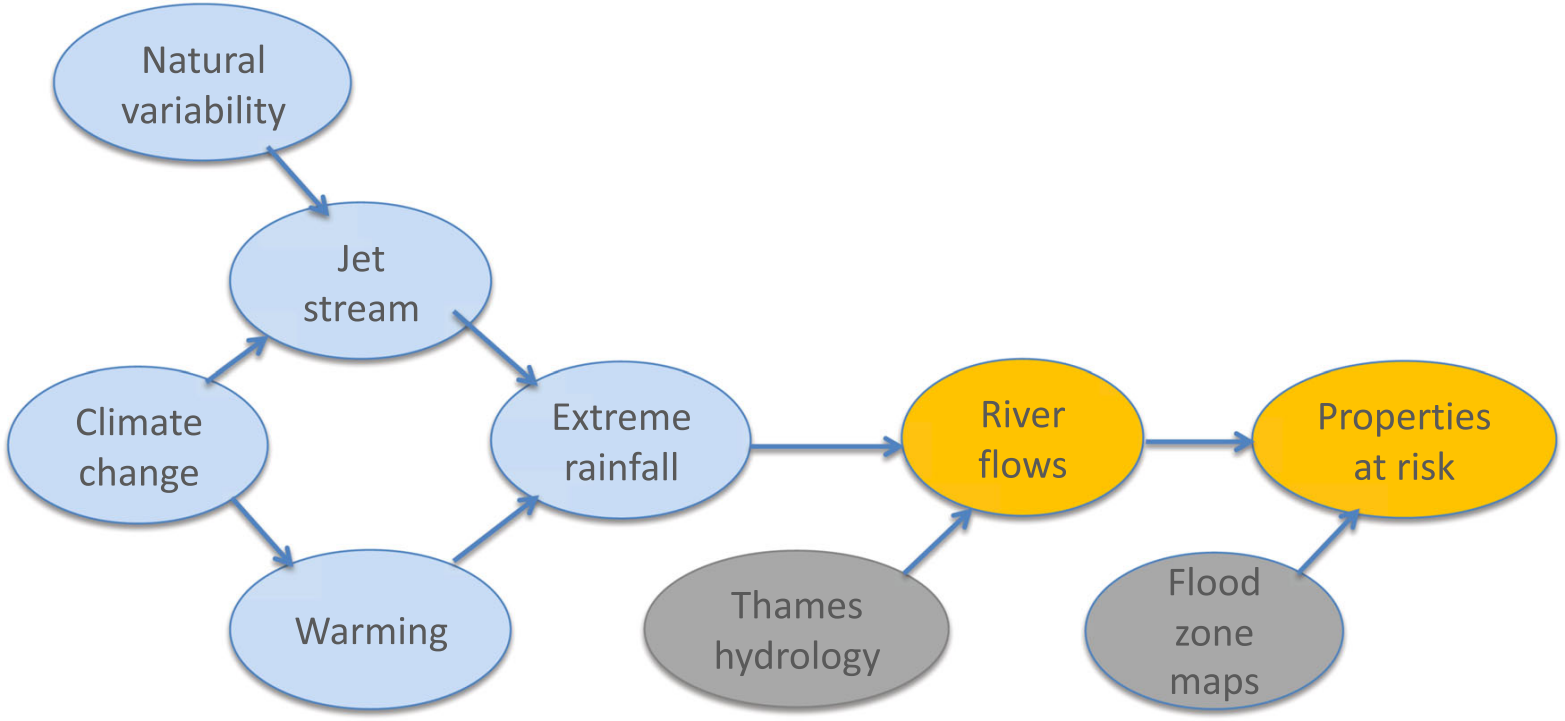
Causal network for discussion of Thames Valley flooding. Arrows indicate the direction of causal influence, but can include the effects of feedbacks. Note that “warming” and “jet stream” are not independent, as they are both affected by “climate change.” The blue shading indicates elements whose causality lies in the weather and climate domain, the gray shading indicates those in the environment and ecosystems domain, and the orange shading indicates a combination of the two. See the text for further details concerning this example.

While the risk‐based research questions can certainly be posed for this extreme event, they are difficult to answer because of the challenges posed by the epistemic uncertainties, which do not have a probabilistic interpretation. This ultimately leads Schaller *et al*.^20^ to employ a conditional wording: “*potentially* at risk” (emphasis added). However, their study makes explicit the various relevant factors, which are represented in Figure 4. All things being equal, a warmer atmosphere holds more moisture, which should lead to more rain; but in the wintertime North Atlantic/European sector, the jet stream is the main determinant of precipitation,^19^ and its response to climate change is highly uncertain. This is reflected in the nonrobust precipitation changes shown in this region in Figure B. The uncertainty propagates through the causal chain, which includes both the hydrology of the Thames River catchment and the details of the exposed properties in the flood zones. This framework lends itself to the storyline research questions. From this perspective, each of the counterfactual calculations, with different jet‐stream responses to climate change, can be treated as different storylines of the event, and the uncertainty in the jet response managed in this way.^21^

## Case studies of extreme events: environmental catastrophes from an ecological and climate perspective

In our discussion in this section of environmental and ecological catastrophes or disasters resulting from extreme weather events, we unite disparate literatures, the biological and the physical. Our goal is to tie together biological understandings of extreme environmental events with physical understandings of extreme weather events into joint causal accounts. This requires fine‐grained descriptions, in both space and time, of the ecological, evolutionary, and biological moving parts of a system together with fine‐grained descriptions, also in both space and time, of the extreme weather events. As Ummenhofer and Meehl^22^ note: “Current‐to‐next generation global climate models, along with higher‐resolution regional models, provide new tools and opportunities for developing a mechanistic, process‐based understanding of where, when and how ECEs [extreme climate events] impact biological systems.” The cultivation of expert‐based rules of thumb (i.e., heuristics) and sophisticated methods for analyzing causes and mechanisms are the goals of ecologically based climate studies. We introduce below a number of preliminary examples of such studies, as instances of what could be pursued in the future in much more detail. In each case, we construct causal network diagrams analogous to that in Figure 4 for the Thames Valley flooding, as a way of depicting how the weather and climate factors interact with the environmental and ecosystem factors to create a complex risk landscape.

A big challenge of performing such studies is to coordinate teams of scientists from both climate and ecological sciences to work on a single, focused event or project. While climate scientists must be concerned with specifying particular extreme climate events, they would simultaneously coordinate with biological scientists working on describing the specific, varied, and sometimes catastrophic consequences of those extreme climate events, thus presenting the reader with a full picture of extreme events and their ecological consequences. Such author teams require flexibility and openness, as well as some sophistication about the complementary scientific fields, no easy set of requirements.

### An Arctic ecosystem collapse

One of the significant predictions of climate change is that low‐lying coastal environments will be flooded by higher sea levels.^23^ In Northwest Canada, the Mackenzie Delta is an ecologically significant ecosystem, long adapted to freshwater flooding during the spring melt of the ice. But when marine (i.e., saltwater) surges penetrate these delta waters during the open‐water season, it can have major impacts on terrestrial and aquatic systems. Pisaric *et al*.^24^ report the examination of both alder tree growth rings and diatoms preserved in lake sediment cores to examine the impacts of a saltwater storm surge in 1999 on the ecosystem of the Mackenzie Delta over the following decade, finding catastrophic major long‐term impacts of this exposure.

The changes in the alder growth rings were dramatic, showing an “abrupt decrease in ring‐width after 1999” (p. 8961 of Ref. 24). There were previously known surge events that affected growth rates in the alders, but they were short‐lived, as opposed to this 1999 event, from which many of the shrubs later died. The changes found in the diatom species distributions from fresh to brackish (mixed salt and fresh water) species were unmatched for over 1000 years of the lake ecosystem. Pisaric *et al*.^24^ surmise that no biological recovery had occurred since the surge event of 1999, and terrestrial vegetation remained drastically changed since the event, which they suggest may mean a permanently altered ecological trajectory. They conclude that as sea ice continues to decline, and similar surge and flooding events occur across other coastal areas in the Arctic, such long‐term and potentially irreversible large‐scale ecological events may be widespread.^24^

Events like this, in which increased storm surge risk from a longer open‐water season and concomitant sea level rise that expose low‐lying Arctic environments to increased surges, such as occurred in 1999, represent impacts not only on the ecosystem at large, but also on the economy and social structure of Arctic communities. Pisaric *et al*.^24^ conclude: “The profound and persistent impact to the terrestrial and aquatic systems suggests that an ecological threshold may have been crossed” (p. 8964). They emphasize that these intrusions of saltwater into previously freshwater ecosystems will also have significant social impacts on nearly all Arctic indigenous communities, which are coastal.

**Figure 5:**
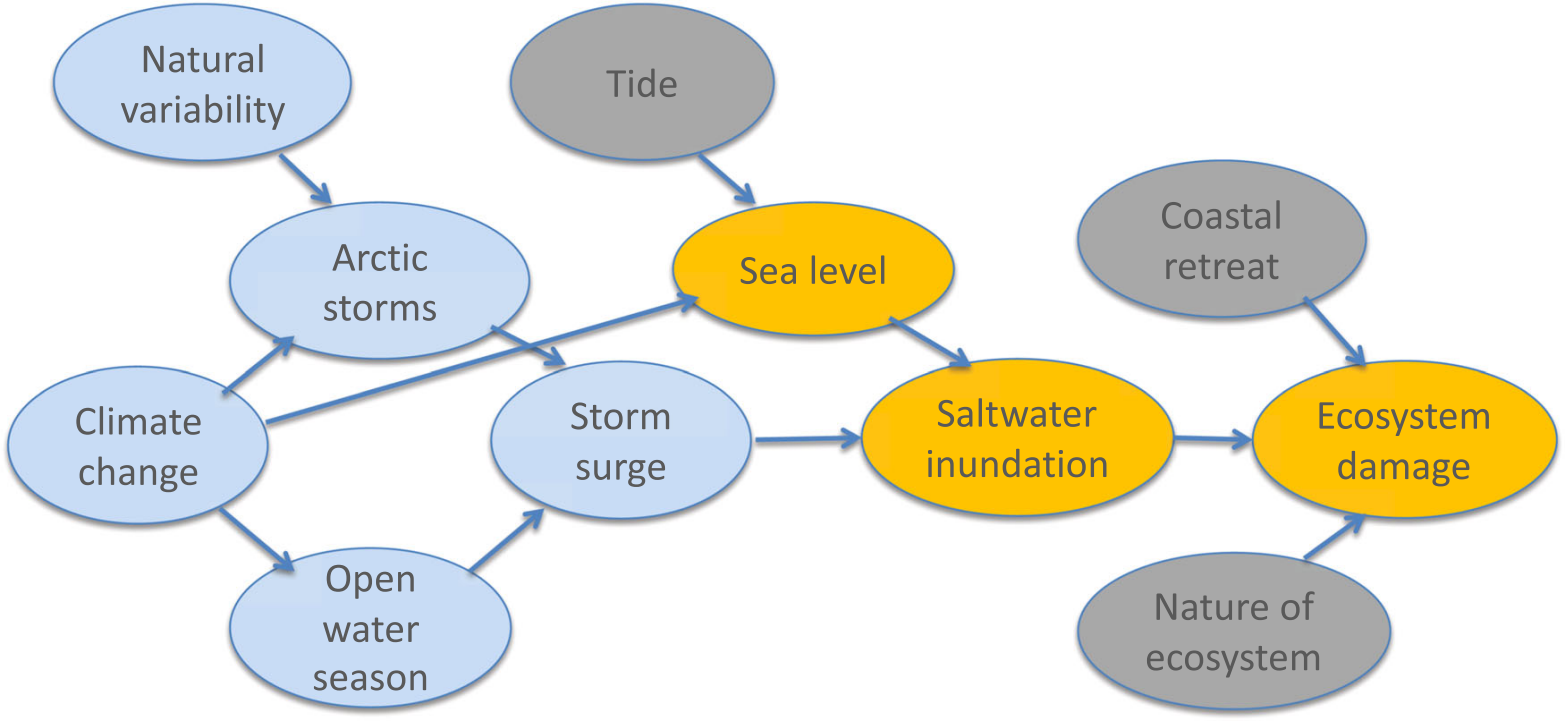
Causal network for discussion of Arctic ecosystem collapse. Arrows indicate direction of causal influence but can include the effects of feedbacks. The blue shading indicates elements whose causality lies in the weather and climate domain, the gray shading those in the environment and ecosystems domain, and the orange shading a combination of the two. See text for further details concerning this example.

The authors do not provide a quantitative attribution to climate change, let alone one expressed in terms of probabilities. Their quantification focuses on demonstrating that the ecosystem event was unprecedented in the 1000‐year‐long historical record and that it was apparently irreversible, that is, it represented an environmental tipping point. From the perspective of attribution, they discuss the various relevant causal factors (represented in Fig. 5), first asking whether they played any role in the 1999 event, and if so, whether there was any plausible link with climate change on the basis of expert knowledge. Their analysis may be represented as follows.

The proximate cause of the ecosystem collapse was the massive saltwater inundation of an ecosystem that was adapted to freshwater flooding. If the ecosystem had been adapted to brackish conditions, then it would not have collapsed. The authors excluded the possibility that the inundation resulted from coastal retreat (which is expected from climate change due to permafrost thawing) and the possibility that the marine flooding was due to a high tide. However, it is known that sea level in this region has risen because of climate change, and that the open water season has lengthened because of sea ice loss.^25^ These are robust, attributed consequences of climate change. Thus, any Arctic storm would, all things being equal, create a storm surge acting on a higher mean sea level and thus lead to greater saltwater intrusion. The authors also appeal to an increase in the intensity of Arctic storms due to climate change, but this assertion is both contestable and unnecessary for the argument. The attribution is expressed largely in qualitative terms, that is, as the risk of saltwater inundation being increased by climate change.

The attribution in this case study is distinctly singular. Observational data are not used to establish probabilities, but to establish the uniqueness of the event for that particular ecosystem. The authors do not argue that climate change will lead to further changes in that ecosystem, since it subsequently adapted to brackish conditions. They argue instead that what happened to that particular ecosystem can be expected to happen widely across the Arctic coast, because the same causal factors will be at play elsewhere, even if to different degrees. They also do not argue that the causal factors that happened to be unimportant in this particular case would be unimportant in other cases. They emphasize the complexity of the causation in that particular situation. In a risk‐based approach, this complexity would present confounding factors that would challenge any definition of what the event actually was, but as discussed above those factors can be independently controlled in a storyline approach. The epistemic uncertainty of how climate change will affect Arctic storms (which are too small scale to be represented accurately in current climate models) would also be problematical for a risk‐based approach but can be managed via a storyline approach.

### A record‐breaking wildfire season

In the record‐breaking 2017 extreme wildfire season in British Columbia, Canada, over 1.2 million ha burned. Human impacts of these fires included displacement of over 65,000 people, as well as impacts on human health and air quality.^26^ Extreme warmth and dryness are regarded as key contributing factors to wildfires, and July–August were anomalously hotter and dryer that year than any other year in the analyzed record, which began in 1961. Using a large ensemble of simulations performed with a regional climate model, Kirchmeier‐Young *et al*.^26^ argue that the wildfires were partially attributable to climate change and made substantially more probable in light of anthropogenic warming. In particular, they concluded that anthropogenic climate change increased the area burned by a factor of between 7 and 11.

Kirchmeier‐Young *et al*.^26^ thus followed the risk‐based approach to attribution. Wildfires are notorious for being difficult to attribute because of the large number of confounding factors, such as forest management, the effect of previous fires, and pests.^2^ These confounding factors make it extremely challenging to detect the effect of climate change in the observational record of wildfires. The authors therefore explicitly ignored those factors and performed a theoretical calculation of fire *risk*, which is to say the likelihood of occurrence all else being equal. In other words, ecological causal factors that are known to be very important for any specific wildfire event were ignored, even though they are likely to have been relevant in this particular event. In that respect, the approach taken was very different from that in the previous section. As a result, the study is less about the attribution of that particular event in the sense of liability, and more about future risk where these ecological confounding factors are highly uncertain and may be regarded as unknown.

Moreover, the calculation of fire risk was not performed with a physically based fire model, but with empirical indices. The main set of indices was from the Canadian Forest Fire Danger Rating System,^27^ which is used internationally, and serves as a predictor of a range of fire weather and behavior characteristics based on weather and climate variables. The indices can thus be considered a form of expert knowledge on the basis of operational experience. The authors also developed their own empirical index of area burned, based on historical data. They were thus able to calculate the change in likelihood of the various indices and area burned as a result of climate change, which led to quantitative statements such as that given above.

**Figure 6:**
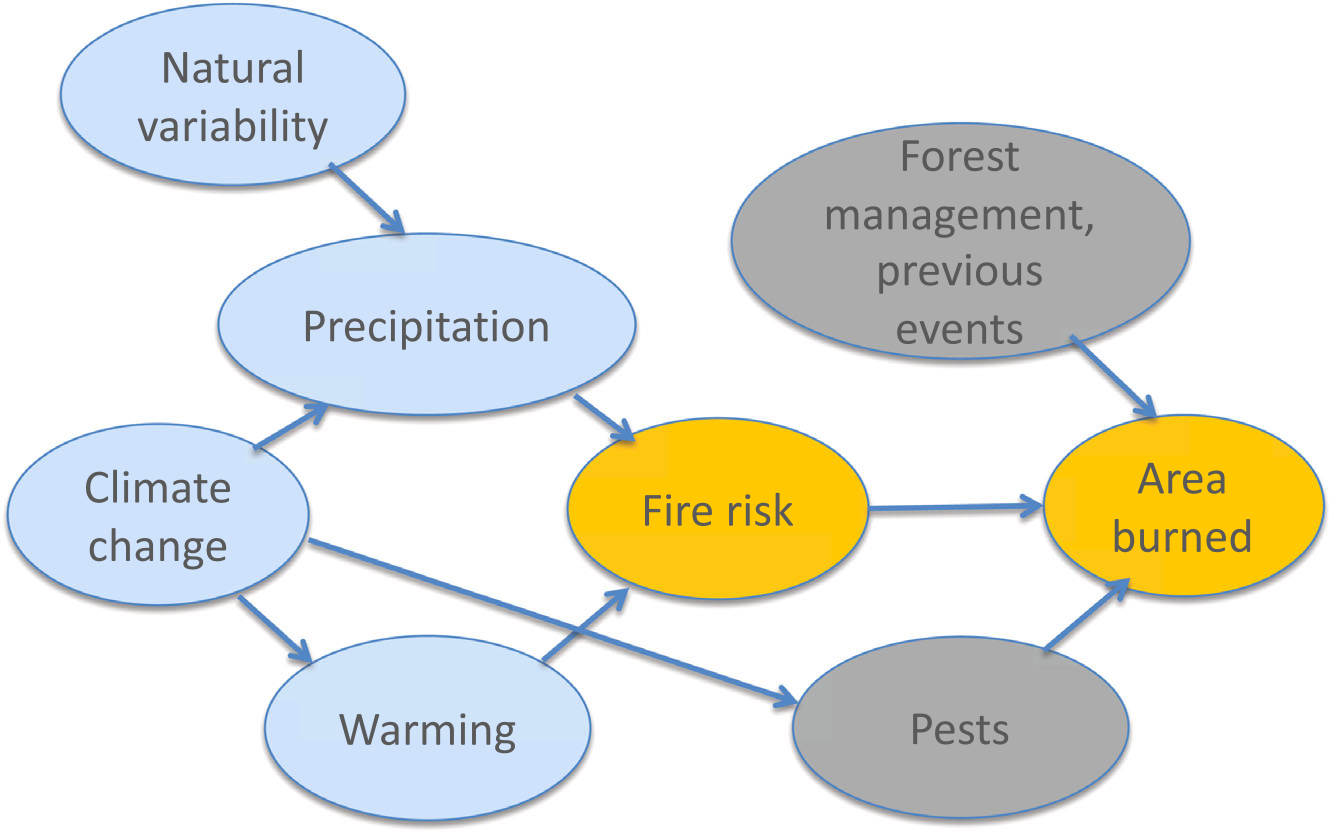
Causal network for discussion of wildfires. Arrows indicate direction of causal influence but can include the effects of feedbacks. The blue shading indicates elements whose causality lies in the weather and climate domain, the gray shading those in the environment and ecosystems domain, and the orange shading a combination of the two. See text for further details concerning this example.

While both dryness and warmth are conducive to fire risk, Kirchmeier‐Young *et al*.^26^ found that in their model simulations, there was no detectable effect of climate change on precipitation in this region. This meant that the high fire risk and area burned that summer inferred in their study was partly due to dryness, which they interpret as natural variability, and partly due to warming, which is a robust consequence of climate change—they estimate the likelihood of the warm conditions to have increased by a factor of 20 because of climate change. For all of the fire indices, “60–90% of the risk … and a factor of 2–4 increase in the likelihood … of the extreme values from 2017 can be attributed to anthropogenic influences” (p. 6 of Ref. 26). However, precipitation can certainly be affected by climate change, and the summertime changes in that region are highly uncertain (Fig. C), with some models projecting more precipitation and some less. As the authors used only a single climate model, and their uncertainties represent only sampling uncertainty, the issue of the unconsidered epistemic uncertainty thus arises here. Their result is most appropriately viewed as a storyline under which the precipitation is assumed not to change (see Fig. 6). If the authors wished to guard against a Type 1 error, they might have considered a storyline with precipitation increasing under climate change, and if they wished to guard against a Type 2 error, they might have considered a storyline with precipitation decreasing under climate change.

A limitation, acknowledged by Kirchmeier‐Young *et al*.,^26^ of any empirical index is that it assumes that the statistical relationship is stationary in time. This can be problematical for applications to climate change. For example, in the year‐to‐year variability on which the statistical model is trained, there will be a correlation between temperature and precipitation (as noted earlier for heat waves), thus precipitation may not be identified as an independent predictor. However, that correlation will almost certainly not be maintained under climate change (especially here since precipitation does not change in their climate model, even though temperature does), implying that precipitation changes represent an independent risk factor. Thus, the ability to apply the risk‐based approach may sometimes come at the cost of having to use empirical relationships that may be questionable.

While we have no specific ecological study of this particular fire season, a significant ecological study of a related fire helps us here. Whitman *et al*.^28^ examined “burn severity,” defined as the “ecological impacts of fire on vegetation and soils,” which was found to influence future stand structure and species composition of the northwestern boreal forest. This study found that land managers use spatial burn severity data to “manage post‐fire risks, ecosystem recovery, and assess the outcomes of fires.” The study assessed burn severity 1 year after the fire in six large wildfires from 2014. Measurements were taken from both standard indices and remote measures, finding consistency with other large fires in North America. The researchers found that weather played an important role in burn severity: “Prognostic models indicated burn severity was explained by pre‐fire stand structure and composition, topoedaphic [soil] context, and fire weather at time of burning” (p. 1 of Ref. 28). Thus, we find that coordinating extreme weather models with burn severity ecological modeling may help us understand the impacts of severe weather events on forest health.

### A single‐year tree die‐off

Moore *et al*.^29^ note that large‐scale tree die‐offs disrupt ecosystem functions and services at major levels. Given that forests cover about 30% of the land surface of the planet and sequester approximately 25% of the carbon dioxide produced by human activities, forest and tree health is globally significant.^30^ With future climate scenarios, drought‐related disturbances may become an important factor in global climate projections through these tree die‐off interventions, changes in tree species compositions, and permanent community changes, because ecosystems that recover from die‐off events pursue different trajectories in successional space, which might be categorized as catastrophic ecological events. Moore *et al*.^29^ focus their attention on the massive tree die‐off from the 2011 Texas drought, the driest year on record for the state, in which many areas reported less than 25% of their usual annual precipitation.^31^ This dramatic reduction in precipitation severely restricted the access to water available to the trees during the summer of 2011. It was also exceptionally warm: the June–August 2011 temperatures were over 1.1 °C higher than the previous record,^31^ exacerbating the stress on the trees. From this drought, it is estimated that approximately 300 million trees died.^29^

To study this event further, Moore *et al*.^29^ investigated widespread tree mortality and their causes and sequelae. The drought‐affected areas spanned mesic to semiarid climate zones, and they sampled nearly 600 0.16 ha plots surveyed in the summer following the drought. In each plot, dead trees were counted that were larger than 12.7 cm in diameter, and identified at the genus level, for 10 regions using remote sensing products of the U.S. Forest Service. Over the state of Texas, regional tree mortality was “massive, with an estimated 6.2% of the live trees perishing, nearly nine times greater than normal annual mortality” (p. 602 of Ref. 29). In addition, most of these trees were larger than the average live tree diameter, “suggesting a re‐ordering of species dominance and downward trend in tree size [that was] most pronounced in the wetter climate zones.” There was drought mortality in more than 29 genera across all regions, which was, surprisingly, equally felt by drought‐resistant and drought‐sensitive species in some regions. The conclusions of the study were that drought‐driven mortality “alters forest structure differently across climate regions and genera” (p. 602 of Ref. 29).

A link to climate change is, however, not straightforward. As Hoerling *et al*.^31^ put it (p. 2811), “Drought and heat are no strangers to Texas,” and Texas is part of a larger regional “warming hole” with no clear manifestation of anthropogenic warming over the 20th century.^32^ The temperature record in this region is confounded by the effects of land‐use change (most famously seen in the Dust Bowl), as drier soils exacerbate heat waves. Moreover, the region is strongly affected by multidecadal natural variability mediated through atmospheric teleconnections driven by sea‐surface temperature patterns in the tropical Pacific Ocean, which can obscure any response to climate change on such timescales.

**Figure 7:**
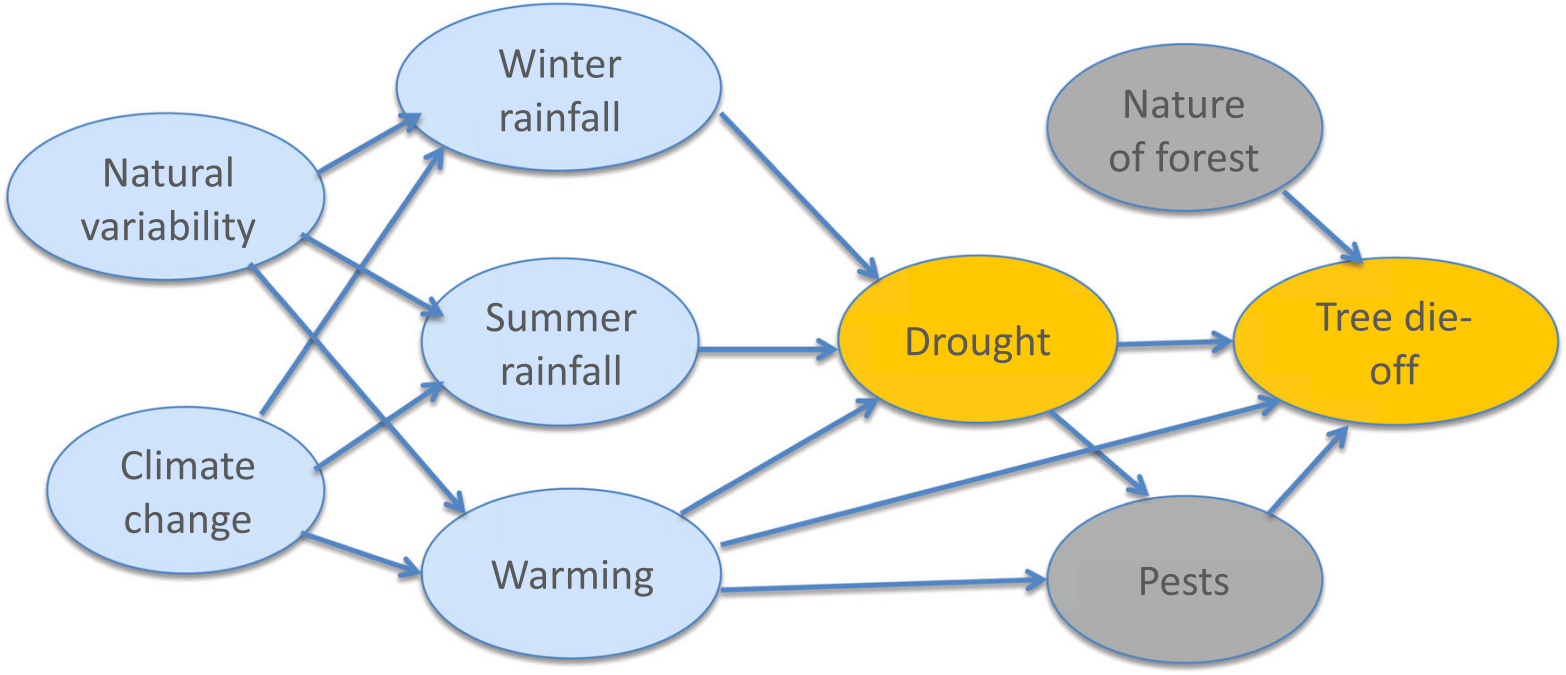
Causal network for discussion of tree die‐off. Arrows indicate direction of causal influence but can include the effects of feedbacks. The blue shading indicates elements whose causality lies in the weather and climate domain, the gray shading those in the environment and ecosystems domain, and the orange shading a combination of the two. See text for further details concerning this example.

Hoerling *et al*.^31^ thus took a storyline approach to the Texas drought event, beginning with the proximate meteorological factors and seeking attribution through the use of a variety of climate model simulations that included informative counterfactual calculations. The role of anomalous sea‐surface temperature patterns in driving the extreme meteorological conditions over Texas, including the precipitation deficit in the winter preceding the drought (which was regarded as a significant causal factor), was of particular focus. The meteorological conditions were represented probabilistically, conditional on the imposed sea‐surface temperature patterns, thus the study illustrates how the storyline and risk‐based approaches can be effectively combined. The various causal factors are represented, together with their impact on the tree die‐off, in Figure 7.

According to the analysis by Hoerling *et al*.,^31^ the unusually dry conditions arose from natural variability, including a very strong component from the large La Niña event that year, which meant the event was predictable in advance. This predictability provides further evidence of causation. They also found that 80% of the heat‐wave magnitude of 2.9 °C was attributable to natural variability (again largely associated with the La Niña conditions), and only 20% of the magnitude, that is, 0.6 °C, to climate change. Given that the tree die‐off was primarily attributed to the drought, this would lead to the conclusion that the die‐off was largely unrelated to climate change.

Following this narrative, while warming temperatures from climate change might be expected to exacerbate such die‐off events in the future, in order to estimate those effects with any precision, it would be necessary to have a detailed understanding of how additional warming would affect the already‐vulnerable and damaged trees left from this drought, which itself would be highly conditional on future natural variability.

However, a key assumption of the narrative of Hoerling *et al*. is that the La Niña conditions that were the main cause of the 2011 drought do not themselves contain a component of climate change. The main evidence for this is that coupled climate models generally show a tendency toward the opposite (i.e., toward El Niño conditions) and furthermore give no clear indication of drying in this region. Yet, the projected precipitation changes in this region are uncertain (Fig. 2); while the multimodel average shown by Hoerling *et al*.^31^ shows essentially no change, this average includes projections with both wetting and drying. Moreover, it has recently been argued that the tendency of current coupled climate models to favor more El Niño‐like conditions reflects shared model biases and is unphysical, and that climate change is likely to favor more La Niña‐like conditions.^33^ As a result, from the perspective of guarding against Type 2 errors, a storyline of increasing drought conditions from climate change should not be ruled out.

### A multiyear tree die‐off

In a review of the causes of drought‐based tree mortality, the authors of Ref. 34 discuss the recovery of hydraulic capacity in trees that survive drought, as well as its failure in those that do not. Recovery of trees after drought is complex, determined by at least the degree of damage to various tissues, the functional status of the remaining hydraulic pathway, the overall health of the remaining foliage and roots, and the water, carbohydrates, and nutrients available during the recovery phase (p. 536 of Ref. 34). They argue that hydraulic failure is the most tractable way to address tree mortality with process‐based models at this time, even though it is known not to be the only way that trees die from drought (p. 537 of Ref. 34).

A related approach is taken by Asner *et al*.^35^ in their study of the California drought of 2012–2015, in which over 200 million trees were lost, including severe impacts on over 58 million large trees.^36^ The authors emphasize a variety of impacts of this millennial‐scale drought on forest and large tree health, to evaluate losses in canopy water content (CWC) of California forests between 2011 and 2015. According to this evaluation, approximately 10.6 million ha of forest experienced measurable loss in CWC during this drought, including severe losses of greater than 30% over 1 million ha. They predict that if drought conditions were to recur or continue, substantial future forest changes would occur.

California forests include the tallest, most massive, and oldest trees on Earth, many of which were killed or damaged by the recent millennial‐scale drought. The authors of Ref. 35 predicted that these losses would have effects on forest fire susceptibility and severity, animal habitat and biological diversity, as well as water resources and carbon sequestration. They took measurements of canopy functional responses to climate change, such as CWC, to improve predictions of how forests would change in the future. CWC is useful because it is an indicator of progressive drought effects on forest canopies, as well as an indicator of vegetation flammability. By using new techniques of aircraft and satellite sensors as well as model‐supported analyses and deep learning algorithms, the authors identify detailed geographic information to support forest management in preparation for climate change.^35^

Asner *et al*.^35^ conclude: “…if drought continues or reoccurs, there exists a pool of trees spread over millions of hectares of forest that may undergo sufficient CWC loss to result in death.… . The findings strongly suggest that if drought continues, even with a potential temporary reprieve via a 2015–2016 El Niño … we can expect continuing forest change at the regional scale” (p. E254). In fact, the 2015–2016 El Niño did bring the expected relief from the drought, and even flooding, but the long‐term impact on the trees’ health of this millennial drought is still unknown.

According to Diffenbaugh *et al*.^37^: “The [California] drought began in 2012 and now includes the lowest calendar‐year and 12‐month precipitation, the highest annual temperature, and the most extreme drought indicators on record. The extremely warm and dry conditions have led to acute water shortages, groundwater overdraft, critically low streamflow, and enhanced wildfire risk” (p. 3931). This is linked to the CWC loss and large tree loss across the state of California.^35^ Other factors associated with tree death and drought may also be considered.^38^ More specifically, the fact that higher specific humidity and increased atmospheric CO_2_ concentrations may actually offset mortality risk from drought and heat^38^, ^39^ means that it may be important to use a causal analysis rather than a correlational approach, since there is no straightforward relationship between increased heat and tree mortality.

The extent to which the California drought can be attributed to climate change has been the subject of much study but remains somewhat open. Much depends on how drought is defined, which is usually motivated by the impacts of interest. For example, meteorological drought is defined in terms of precipitation deficit, agricultural drought in terms of soil moisture deficit, and hydrological drought in terms of water shortage. As in many other regions of the world, the precipitation response to climate change in California is highly uncertain (Fig. 2), but warming is robust. Standard indices for drought, such as the Palmer Drought Severity Index, which combines precipitation deficit and temperature, therefore tend to suggest a robust increase in drought risk from climate change, driven by the warming.^37^, ^40^ The relevance of temperature for drought is very context specific, so such coarse‐grained indices are only indicative, but more detailed analysis has confirmed that warming has been a significant contributor to the recent California drought severity,^41^ and more generally is expected to exacerbate drought across the Southwest and Central Plains of Western North America.^42^

The storyline analysis of Diffenbaugh *et al*.^37^ clearly delineates the roles of increasing greenhouse gases, natural variability, and the interactions of these factors to produce the very unusually dry and hot weather that produced the extraordinary stress on the trees during the multiyear California drought. They emphasize the role of a long‐term blocking pattern: “The proximal cause of the precipitation deficits was the recurring poleward deflection of the cool‐season storm track by a region of persistently high atmospheric pressure, which steered Pacific storms away from California over consecutive seasons” (p. 3931 of Ref. 37). Although Diffenbaugh *et al*.^37^ cite a few studies that suggest these blocking conditions might become more frequent under climate change, their overall conclusion is more agnostic, given the lack of a clear precipitation trend in the historical record. It might be added that the response of atmospheric blocking to climate change is highly uncertain^43^ and that climate models generally predict, if anything, increased wintertime wetting in this region (Fig. B; see also Ref. 44). Thus, different storylines of precipitation change might be reasonably considered. The extent to which warming will exacerbate drought would be conditional on such storylines.

Returning to the causal relationships between drought and tree die‐back (Fig. 7), again, these are not straightforward and may be complicated by other interacting factors, such as fire and wind. Moreover, drought and heat stress “can significantly amplify the incidence and severity of biological disturbances such as outbreaks of damaging insects and diseases” (p. 15 of Ref. 38). The authors of Ref. 38 (p. 15) call for a “better mechanistic representation of the diverse processes that drive tree mortality under drought,” which they view as necessary for improving predictions of forest responses to future climate change.

Ummenhofer and Meehl^22^ emphasize that “Droughts are most likely to have the largest and most long‐lasting impacts” among extreme climate events, “globally due to large indirect and lagged impacts and long recovery especially for forest ecosystems.” They offer as examples the subcontinental die‐off of woody plants during the early 2000s in the American Southwest that killed 90% of the dominant pine species, as well as the 2005 drought in the Amazon ecosystem that was so severe that it reversed the forest's role as a long‐term carbon sink.^22^

### Crossing ecosystem tipping points in Australia

Harris *et al*.^45^ use the “press and pulse” framework, applied in ecological systems for many years, to understand how climate and climate change impinges on biological species and communities in Australia: they use this ecological framework “to explain potential ecosystem responses to long‐term changes in climate trajectories (presses) and extreme events (pulses)” (p. 579). In our view, the “press” in this framework maps well onto the thermodynamic (warming) aspects of climate change, while “pulse” maps onto dynamic and extreme aspects, including drought. As Harris *et al*.^45^ note, “Although often considered separately in both climate models and biological experiments, in reality presses and pulses, exerted simultaneously, may be more likely to push systems to tipping points” (p. 579; see also Ref. 46), which are described by Scheffer *et al*.^47^ as “catastrophic shifts in ecosystems.” Indeed, most assessments of extreme events using climate models consider the warming trend together with the dynamical variability and can potentially be used to examine presses and pulses in combination.

Harris *et al*.^45^ argue that the long‐term effects of extreme weather events may lead to “community‐level responses such as changes in species richness, composition and/or dominance,” and even local or species‐wide extinction.^45^, ^48^, ^49^ Moreover, these changes may be long lasting or irreversible, especially if stabilizing ecological feedbacks are changed, or repeated extreme events occur. Such feedbacks may, for example, include predation, competition, or ecological facilitation.^45^, ^50^, ^51^, ^52^

Because the magnitude and frequency of many extreme weather events (relative to the historical baseline) is expected to increase with climate change,^3^ the threshold between extinction extremes and survivable weather events is likely to be crossed more frequently. Climate change having influences of this kind can prevent recovery of a population or species after an extreme event, with long‐term consequences for population size and persistence of the species.

Because organisms are adapted to local levels of climate variability, the size of the deviation from the mean has the greatest biological impact. Some extreme events are described in terms of an absolute threshold, for example, number of days above a certain temperature, but they are also frequently defined in relative terms. For biological applications, extreme events are often operationally defined as falling outside the 10th or 90th percentile of the probability density function on the basis of historical observations.^45^, ^53^ Even small shifts in the distributions of climate variables can result in major changes to the frequency and magnitude of extreme events, as we reviewed above, as can changes in the variance or shape of the distribution.

The intensity, frequency, and duration of heatwaves in Australia have increased since 1950, although this varies by location, and record‐breaking warm events outnumber record‐breaking cold events 12 to 1 (p. 582 of Ref. 45). As the temperatures go up, fire danger increases, with weather conditions that are conducive to extremes of fire danger and the extension of the fire season since the 1970s.

We have reason to think that Australia's flora and fauna have been affected by recent extreme events, because several recent attribution studies, using the risk‐based approach, have detected increases in the likelihood of specific observed extreme temperature events on the continent, attributable to climate change.^54^

Australian ecosystems are well adapted to interannual and interdecadal climate variability; however, the recent rise in temperatures and change in precipitation patterns have imposed new demands on biological systems not seen before. As the interval between extreme events decreases, there has been an increase in negative impacts on biodiversity in Australia, as exhibited by the six case studies shown in Ref. 45, which “collectively … demonstrate how the ongoing press of climate change can lead to ecological catastrophe given climatic pulse [extreme] events at critical periods” (p. 583).

Harris *et al*.^45^ note that “a single extreme event can be sufficient to cause irreversible regime shift or an ecosystem ‘tipping point’” (p. 583), giving the example of the kelp forest regime shift of south‐western Australia. It is attributable to a single heatwave (pulse) superimposed on the pattern or press of increasing sea‐surface temperatures over a long period. Harris *et al*.^45^ describe the event this way: “Summer ocean temperatures between 2011 and 2013 were the hottest in over 140 years. The warmest year was 2011, with temperatures 2 and 5 degrees C above the long‐term mean, extending over 2,000 km of coastline for more than 10 weeks. This event led directly to mortality in kelp, abalone, coral, fish and lobster populations” (pp. 583–584). This single die‐back led to a permanent range contraction in the kelp forest, which resulted from competition with turf seaweed and grazing by tropical fish species, which were patterns established during the heat wave.

A different ecological catastrophe also demonstrated the damage that extreme events could do, especially when they recur over ever‐decreasing periods, which can lead to population collapse if the population does not have time to recover before the event recurs.^55^ In Australia, many wildfires in short succession, which arose from dangerous fire weather, resulted in the conversion of the habitat of obligate seeder “*Eucalyptus delegatensis* forest to shrubland in the Australian Alps, a process that potentially threatens the entire species’ range” (p. 584 of Ref. 45), potentially leading ultimately to extinction.

**Figure 8:**
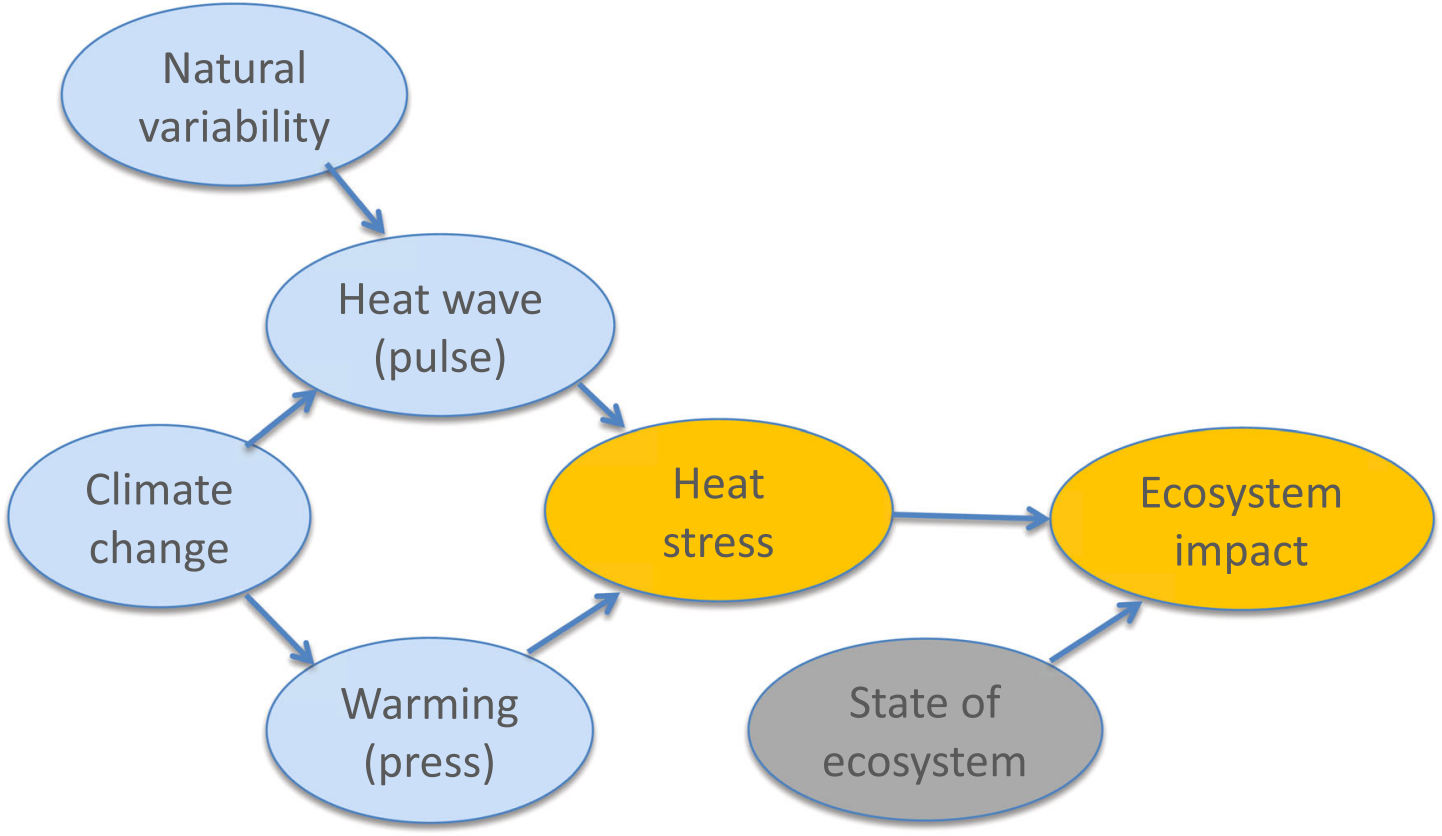
Causal network for discussion of heat stress–driven ecosystem tipping points. Arrows indicate direction of causal influence but can include the effects of feedbacks. The blue shading indicates elements whose causality lies in the weather and climate domain, the gray shading those in the environment and ecosystems domain, and the orange shading a combination of the two. See text for further details concerning this example.

These case studies of Harris *et al*.^45^ represent something like a meta‐analysis of storyline approaches, with causal factors as represented in Figure 8. The driving climatic factor in these cases is mainly the higher temperatures, which is a robust outcome of climate change and thus could be amenable to a risk‐based approach. However, it is clear from the details of the case studies that mapping the warming onto the specific ecosystem tipping point in a probabilistic way would be extremely challenging.

## Summary and ways forward

The various case studies discussed above illustrate that the probabilistic, risk‐based approach to extreme event attribution, which generally asks largely unconditional questions about changes in likelihood or magnitude of the event arising from climate change (e.g., Fig. 1), is difficult to apply meaningfully to environmental and ecosystem events. Difficulties arise from the necessarily coarse‐grained definition of the weather event (required in order to achieve a large sample size) and the sensitivity of the research answer to how that coarse graining is done. This sensitivity would only be amplified by the spatiotemporal downscaling that would be required to translate the extreme weather event into environmental or ecosystem impacts, given the strong nonlinearity of those impacts. A second difficulty arises from the large number of confounding factors that, given the inevitable data limitations, cannot possibly be controlled for using conventional statistical approaches, such as regression. As a result, the conclusions almost necessarily must ignore such factors, even though they lie at the heart of environmental and ecological concern, and indeed typically relate to policy and management options. A final difficulty arises from the inability to represent epistemic uncertainties in a probabilistic fashion, which even arises at the level of the weather extreme itself and would again only be amplified at the impact level. Together, these difficulties mean that the conventional “falsificationist” approach, which aims to define scientific knowledge by excluding incorrect hypotheses, and thus preferentially aims to guard against Type 1 errors, is ill suited to this scientific challenge.

Our case studies have illustrated that a more fruitful approach to understanding and quantifying the role of climate change in extreme environmental and ecological events is the storyline approach, which asks singular questions about the causal factors that were relevant in a particular event, and how those factors may have changed and could change in the future. Thus, it begins from the knowledge of where we are, anchored in data and process understanding, and explores sensitivities to that state. The answers to these more forensic kinds of research questions are represented in a highly conditional form, as illustrated in the causal network diagrams provided for each case study and the accompanying narratives. The storyline approach is a rather Bayesian way of constructing scientific knowledge,^21^ and allows a layered approach to attribution, which can be tailored to the level of understanding that exists in a particular situation and sharpened as knowledge improves. It is also well designed for guarding against Type 2 errors, which is often the perspective of conservation management.

Harris *et al*.^45^ urge the study of pulse and press events and how the pulses and presses interact with each other in order to understand how they drive abrupt or catastrophic ecological change. They believe that such study may improve our ability to detect climate change impacts on biological systems, as well as improve the understanding of how ecological processes respond to extreme weather events and climate change. But studies such as these must rely on adequate survey methods and instruments, some of which are only now being developed or put online.

Earlier, Jentsch *et al*.^56^ had concluded that long‐term observations and experimental studies in different ecosystem types and at distinct spatial and temporal scales are crucial for understanding the impact of extreme weather events on ecological systems. The fact that nearly all existing biological and ecological records at that time were local and site‐specific meant that such information could not be usefully combined with more coarse‐resolution climate models without downscaling first. When looking at extreme weather events, the problem is worse, because such events are, almost by definition, rare, and to sample a distribution in such a way as to license inferences about its tails, long time series are needed. Thus, comprehension of the necessary biological/physical interactions is needed, we reiterate, for understanding and predicting how extreme weather events influence ecological systems.

There is much promise in the increasing resolution of remote sensing networks of environmental, biological, and climate conditions at compatible spatial and temporal resolutions.^22^, ^57^, ^58^ Examples would include monitoring of soil properties, concurrent vegetation states, such as biomass, and radiative properties, such as fractions of absorbed radiation.^22^, ^59^ With remotely sensed properties, impacts on plant physiology, photosynthesis, damage to trees, and topsoil erosion and its effects can be measured, “as well as lagged impacts like changes in plant phenology, reduced plant growth, increased mortality and changes in plant species composition” (p. 8 of Ref. 22; see also Ref. 59).

As we learn more about how climate change affects extreme weather events, including what triggers them as well as their local contexts, it should, in principle, become easier to better inform mitigation and adaptation efforts. But the complexity of the interactions between long‐term climate change (press) and shorter‐term extreme events (pulse), and how these are imprinted on the biology of organisms, means that it is very hard to take any quantification of risks of extreme events and translate it into meaningful terms of biological risks and their response to such extreme events. This lessens our options of being able to mitigate or adapt to them early in the process before more damage occurs. As noted above, a causally based storyline approach is more promising, although still daunting.

There are ways, however, to better predict ecological responses to extreme weather events. Harris *et al*.^45^ suggest that ecosystem models, currently calibrated for equilibrium conditions, “would be greatly improved by incorporating a better mechanistic understanding of the impacts and responses to interacting climate presses and pulses” (p. 585). Similarly, Parmesan *et al*.^60^ saw opportunities for advances in ecological and evolutionary theory, such as population dynamics, physiological energetics, and community structure, which could lead to increased predictive power if better ability to predict the likelihood of extreme weather events were combined with better representation of biological parameters. They focused on the complex interactions between climatic conditions and biological systems spanning the interactions’ entire spatial and temporal spectra. Parmesan^61^ rues the lack of mechanistic understanding relating climatic and biological systems, arguing that developing process‐based concepts of biological responses to extreme weather events is crucial for predicting ecosystem impacts in the future. We offer this paper as a brief collection of such cases, pulling together threads from disparate biological and climate literatures to tell a coordinated story of ecological catastrophe and extreme events. As we learn how organisms adapt and evolve in response to extreme climate challenges, this will help our ability to predict environmental futures under such extremes.

Presumably, deeper understanding of such biological responses will come with increased measurement and study of biological parameters; the advantages of remote sensing should soon be felt theoretically as well as empirically. It is generally agreed that meta‐analyses (e.g., Refs. 62 and 63) can advance the field in lieu of detailed quantitative formal risk‐based attribution studies. We would like to add that storyline approaches make sense of these accounts, which combine observations, experiments, remote sensing, and climate model output, and can provide, in some cases, causal factors useful for predicting biological impacts of extreme weather events. As shown in Ref. 45, the press–pulse framework helps frame the research questions into biologically meaningful terms, and we note that there is a natural mapping of press onto the thermodynamic (warming) aspects of climate change and of pulse onto the dynamic and extreme aspects, including natural variability. Better understanding of anything that would increase or decrease the impact of an extreme weather event will help predict the outcome of such a future event, even if the likelihood of the event cannot be confidently predicted. Such a form of conditional knowledge corresponds to what Mastrandrea *et al*. call a conditional finding and is a well‐accepted form of knowledge within the IPCC Working Group II landscape.^11^

Such improved knowledge of the effect of climate change extremes or pulses on ecological systems, in turn, might promote better mitigation and adaptation strategies. In conservation biology, such strategies might be controversial, as little is known about ecological catastrophes induced by climate‐related extreme weather events worldwide. However, generally, extreme biological responses, according to Harris *et al*.,^45^ “are likely to be characterized by abrupt ecological changes and long recovery times” (p. 585). Moreover, they can be highly nonlinear, especially as compared with responses to the average effects of climate change, or press. But as the frequency of extreme events increases, and there is less recovery time between extreme events, the scope for conservation‐aimed interventions becomes less possible and less promising. The extreme responses generated by extreme weather events “call for greater policy and philosophical fluidity in conservation management, greater capacity and appetite for interventions, and detailed documentation of the consequences of interventions” argue Harris *et al*.^45^ (p. 584). They note that the needed interventions may involve practices that are not widely accepted, such as “Assisted colonizations, … or the translocation of warm‐adapted genotypes” (p. 584). They conclude that “The risk of nonintervention may outweigh the risk of intervention more often in the future” (p. 584), a claim that surely will be debated among ecologists and conservation biologists of today and into the future. While we do not endorse the detailed conclusions of Harris *et al*.,^45^ they raise some key questions that directly relate to the Type 1/Type 2 error dichotomy we have highlighted: Will the risk of nonintervention outweigh the risk of intervention? Is there any alternative?

## Competing interests

The authors declare no competing interests.
